# Promoting presumptive diagnosis of severe HIV disease to increase uptake of antiretroviral therapy in HIV-infected infants

**DOI:** 10.1186/1758-2652-13-S4-P155

**Published:** 2010-11-08

**Authors:** J Chiwoko, E Mhango, S Phiri, R Weigel

**Affiliations:** 1 Kamuzu Central Hospital, Lighthouse Trust, Lilongwe, Malawi; 2 Ministry of Health, HIV department, Lilongwe, Malawi

## Purpose of the study

Despite successful scale up of anti-retroviral therapy (ART) in Malawi, the number of children initiating ART early in their life is low and mortality of HIV infected infants not accessing ART is high. Availability of PCR remains limited and despite presumptive diagnosis (PD) is recommended in the national guidelines, it is rarely used as a reason to start HIV-exposed infants on ART. Reasons may include factors at health worker and clinic level. Knowledge of these barriers is important to increase access to ART for this high risk group.

## Methods

A structured questionnaire with 11 closed questions was administered to one ART clinic staff member for each site (nurse or clinician) by team-members of the regular national ART supervision for Quarter 1/2009 in the central and southern region. Questions addressed specific reasons for low uptake of PD and tested providers' knowledge of the PD definition. Finally, the interviewer explained how to make a PD correctly using a job-aid.

## Results

Over 2 weeks, respondents of 49 of 173 ART-sites completed the questionnaire. They were mainly clinical officers (22) and nurses (17) from a central hospital, 5 district hospitals, 3 rural hospitals, 13 health centers, and 27 other facilities such as mission hospitals, smaller clinics run by NGOs, armed forces or privately. Forty-one clinics provided HIV testing and counseling for pregnant women on-site and 35 clinics offered antiretrovirals for PMTCT. Most providers felt that children <18 month of age are under-represented in their ART-cohort, mentioned that generally few HIV exposed children are eligible for ART in their catchment area and that limited access to PCR are reasons for low uptake of PD (34/49 respondents each). Twenty-nine saw the presence of maternal HIV-antibodies as a barrier that makes the diagnosis difficult, and 20 noted that staff at the under 5 clinic or postnatal ward rarely identifies and refers HIV exposed infants to HIV-services. Twelve providers said they generally don't register children at that age. Of the 49 providers, 15 defined presumptive diagnosis correctly and 15 partly correct.

## Conclusions

Providers seem to be aware of the need to start more HIV-infected infants on ART and of structural barriers to access this service. Improved knowledge and sensitization of providers on how to diagnose severely ill HIV-exposed infants presumptively may increase access to ART for children in this age-group.

